# The discrepancy between radiographically-assessed and self-recognized hallux valgus in a large population-based cohort

**DOI:** 10.1186/s12891-021-04978-z

**Published:** 2022-01-04

**Authors:** Takumi Matsumoto, Junya Higuchi, Yuji Maenohara, Song Ho Chang, Toshiko Iidaka, Chiaki Horii, Hiroyuki Oka, Shigeyuki Muraki, Hiroshi Hashizume, Hiroshi Yamada, Munehito Yoshida, Kozo Nakamura, Sakae Tanaka, Noriko Yoshimura

**Affiliations:** 1 Department of Orthopaedic Surgery, Faculty of Medicine, The University of Tokyo, 7-3-1 Hongo, Bunkyo-ku, Tokyo, 113-8655 Japan; 2 Department of Preventive Medicine for Locomotive Organ Disorders, 22nd Century Medical & Research Center, Faculty of Medicine, University of Tokyo, Tokyo, Japan; 3 Department of Medical Research and Management for Musculoskeletal Pain, 22nd Century Medical & Research Center, Faculty of Medicine, University of Tokyo, Tokyo, Japan; 4 School of Health and Nursing Science, Wakayama Medical University, Wakayama, Japan; 5 Department of Orthopaedic Surgery, Wakayama Medical University, Wakayama, Japan; 6 Sumiya Orthopaedic Hospital, Wakayama, Japan; 7 Towa Hospital, Tokyo, Japan

**Keywords:** Hallux valgus, Hallux valgus interphalangeus, Prevalence, Radiograph, Self-recognition

## Abstract

**Background:**

There has been a paucity of literature revealing the discrepancy between self-recognition about hallux valgus (HV) and radiographically-evaluated foot configuration. Knowing this discrepancy will help to make a comparative review of the findings of previous literatures about epidemiological studies about the prevalence of HV.

**Questions/purposes:**

(1) Is there a discrepancy between radiographically-assessed and self-recognized HV in the general population? (2) What factors affect the self-recognition of HV in the general population?

**Methods:**

The fifth survey of the Research on Osteoarthritis/Osteoporosis against Disability study involved 1996 participants who had undergone anterior-posterior radiography of bilateral feet and answered a simple dichotomous questionnaire on self-recognition of HV. Measurements of the HV angle (HVA), interphalangeal angle of the hallux (IPA), and intermetatarsal angle between 1st and 2nd metatarsals (IMA) were performed using radiographs. Radiographic diagnosis of HV was done using the definition of hallux valgus angle of 20° or more. After univariate comparison of the participant backgrounds and radiographic measurements between participants with or without self-recognition of HV, multivariable logistic regression analysis was conducted in order to reveal independent factors affecting self-recognition.

**Results:**

Significant difference was found between the prevalence of radiographically-assessed and self-recognized HV (29.8% vs. 16.5%, *p* <  0.0001). The prevalence of self-recognized HV increased with the progression of HV severity from a single-digit percentage (normal grade, HVA < 20°) up to 100% (severe grade, HVA ≥ 40°). A multivariable logistic regression analysis demonstrated that HVA, IMA, and female sex were independent positive factors for self-recognition of HV (HVA [per 1° increase]: OR, 1.18; 95% CI, 1.15–1.20; *p* <  0.0001; IMA [per 1° increase]: OR, 1.15; 95% CI, 1.09–1.20; *p* <  0.0001; and female sex [vs. male sex]: OR, 3.47; 95% CI, 2.35–5.18; *p* <  0.0001).

**Conclusions:**

There was a significant discrepancy between radiographically-assessed and self-recognized HV which narrowed with the progressing severity of HV. HVA, IMA, and female sex were independent positive factors for self-recognition of HV. Attention needs to be paid to potentially lowered prevalence of HV in epidemiological studies using self-reporting based on self-recognition.

## Background

Hallux valgus (HV) is a common foot deformity associated with a laterally-deviated hallux and a medially-prominent first metatarsal head [1]. HV causes pain and difficulties in finding properly fitted footwear, and correlates with functional disability and elevated risk for falls in elderly adults [2, 3]. A pooled analysis on HV prevalence provided an estimate of 23% in the general population aged between 18 and 65 years [4]. While differences in race, footwear habits, and regions have been known to affect the true prevalence of HV [5–7], the reported prevalence is also affected by research methods, including sampling methodology, study quality, and method of HV diagnosis. For example, a systematic review of the literature on HV prevalence demonstrated that the studies reporting HV prevalence by means of clinical examination showed a higher prevalence than those that used self-reporting with interviews or questionnaires [4]. Several approaches other than a simple dichotomous question have been suggested, including the use of standardized photographs or line drawings, in order to make up for the shortcomings of self-reporting [8, 9]. However, there has been a paucity of literature revealing the discrepancy between self-recognition about HV and radiographically-evaluated foot configuration in the same cohort. Knowing the gap between self-recognition about HV and actual deformity in the general population will not only provide a better interpretation of previous surveys on HV prevalence, but will also help to construct strategies for preventing HV. Therefore, the present study was aimed for investigating the relationship between HV self-recognition and radiographically-assessed hallux deformity, and to clarify the factors influencing self-recognition of HV in the general Japanese population.

## Patients & methods

### Study design

**Fig. 1:**
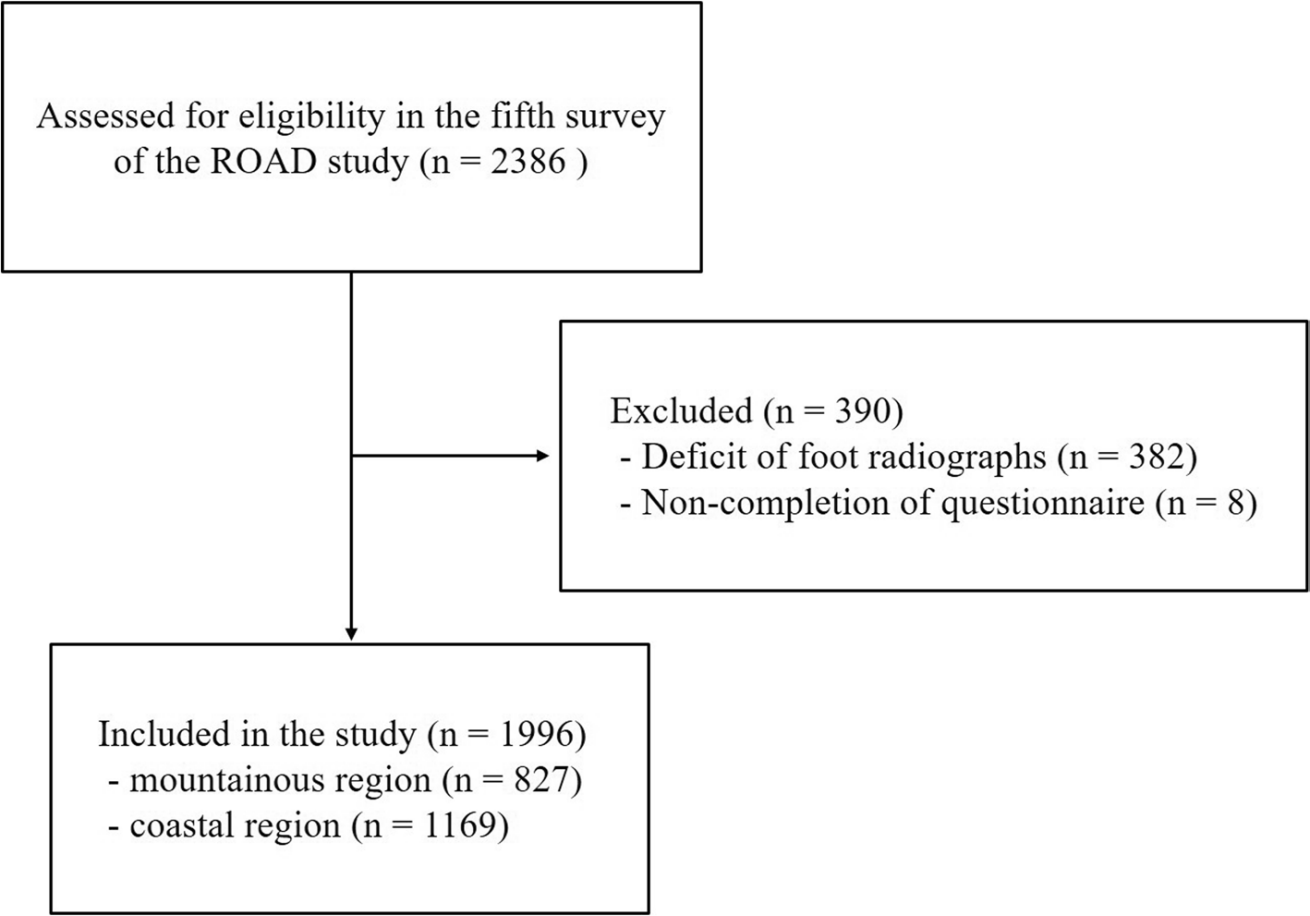
Study flowchart. ROAD; Research on Osteoarthritis/Osteoporosis Against Disability

The present study was performed as part of the fifth survey of the Research on Osteoarthritis/Osteoporosis Against Disability (ROAD) study. The details of the ROAD study have been previously described [10]. It is a nationwide prospective study established in 2005 aiming for aggregating the epidemiological data about musculoskeletal disorders, especially osteoarthritis (OA) and osteoporosis. It consists of three local residents cohorts from the following three different regions in Japan: an urban region (Itabashi district, Tokyo); a mountainous region (Hidakagawa city, Wakayama); and a coastal region (Taiji city, Wakayama). The resident registration lists of the communities were used for recruitment of the participants. The fifth survey was performed during 2018–2019 in the mountainous and coastal regions with 2386 participants. A total of 827 subjects in the mountainous region and 1169 participants in the coastal region had radiographs of both feet and had answered interviewer-administered questionnaires; therefore, their data were used for analysis (Fig. 1). The interviewer-administered questionnaire consisted of information regarding the participants’ personal medical history, family history, physical activity, and pain in the joint, among others. With respect to the foot, the participants were asked if they thought they had HV as a dichotomous question of yes or no, separately for their left and right feet. An equivalent term in translation for HV in Japanese was used for questionnaire first; however those who did not understand the meaning of HV were asked if they thought their halluces were deviated laterally instead. The participants who answered yes to this question were further interviewed on the following items: 1) since when they had been aware of having HV; 2) whether their HV was accompanied by pain; and 3) if there was a history of hospital visit for HV.

### Radiographic assessment

Anterior-posterior radiographs of the bilateral feet of each participant were obtained in the supine position with the plantar aspect of their feet resting on the image receptor and with the beam angled approximately 15° posteriorly towards the calcaneus, by licensed radiography technicians. The HV angle (HVA), the interphalangeal angle of the hallux (IPA), and the intermetatarsal angle between 1st and 2nd metatarsals (IMA) were measured in the foot radiographs as the angle between two longitudinal axes of the first metatarsal and the proximal phalanx [11], the angle between two longitudinal axes of the proximal phalanx and the distal phalanx [12], and the angle between the first and second metatarsals [11], respectively. All of the radiographic evaluations were digitally measured with OsiriX software (Pixmeo SARL, Bernex, Switzerland). All measurements were achieved by an independent orthopedist who was a board-certified member of the Japanese Orthopedic Association in order to eliminate the influence of inter-observer variability. The grade of HV severity was classified using the HVA, according to the Japanese Orthopedic Society criteria, as follows: < 20°, normal; 20–29°, mild; 30–39°, moderate; and ≥ 40 °, severe [13]. The total valgus deformity of the hallux (TVDH) was figured by adding the HVA and IPA [12].

In order to evaluate the precision of measurements, the intraclass correlation coefficient (ICC) and the standard error of measurement (SEM) were calculated using 35 randomly selected films using random number generator software program. Evaluations were performed two times at an interval of 6 weeks blinding the patients’ identity and previously recorded values. The ICCs for the HVA, IPA, and IMA were 0.99, 0.97, and 0.97, respectively. The SEMs for the HVA, IPA, and IMA were 1.0 degrees (95% CI, 0.7–1.2), 1.4 degrees (95% CI, 1.1–1.6), and 0.9 degrees (95% CI, 0.6–1.1), respectively. These results indicated good reproducibility and validity of the measurements.

### Statistics

Continuous variables were expressed as mean ± standard deviation. Proportions were presented as counts and percentages. Pearson’s correlation coefficients were applied to examine the relationship between the HVA and IPA. Comparisons of participants’ backgrounds and radiographic parameters between groups (men vs. women, or participants with self-recognition about HV vs. those without) were performed using appropriate statistical methods as described below. The Student’s t-test was used for comparison of continuous variables. The chi-square test or Fisher’s exact test was used for comparison of proportions between groups followed by adjusted residual analysis in cases with significant differences. Multivariable logistic regression analysis was performed to clarify the independent factors influencing self-recognition of HV. All variables having a *p*-value of less than 0.05 in univariate analysis were put into the model. Statistical differences were considered significant when a p-value was less than 0.05. The JMP12 statistical software (SAS Institute Inc., Cary, NC, USA) was used for all statistical analyses.

## Results

The participant characteristics were summarized in Table 1. In the whole cohort, the percentage of participants who had at least one radiographic HV was 39.0%, with a significant difference between men and women (22.8% vs. 46.0%, *p* <  0.05). Bilateral HV was detected in 423 participants (21.2%), with a significant difference between men and women (10.2% vs. 26.5%, *p* <  0.05).

**Table 1:** Participants’ characteristics and comparisons between men and women

	Total (*n* = 1996)	Men (*n* = 654)	Women (*n* = 1342)	*P*-value
Age (years)	64.2 ± 12.7	63.9 ± 13.7	64.4 ± 12.1	0.35
Age distribution (n [%])				
< 39	64 (3.2%)	29 (4.4%)^a^	35 (2.6%)	< 0.0001
40–49	191 (9.6%)	72 (11.0%)	119 (8.9%)	
50–59	348 (17.4%)	102 (15.6%)	246 (18.3%)	
60–69	593 (29.7%)	193 (29.5%)	400 (29.8%)	
70–79	555 (27.8%)	165 (25.2%)	390 (29.0%)	
80≦	245 (12.2%)	93 (14.2%)	152 (11.3%)	
Height (cm)	158.0 ± 9.1	167.1 ± 6.7	153.5 ± 6.7	< 0.0001
Body weight (kg)	57.0 ± 11.6	66.0 ± 11.1	52.6 ± 9.0	< 0.0001
Residing in the coastal area	1169 (58.6%)	377 (57.7%)	792 (59.0%)	0.56
Body mass index (kg/m^2^)	22.7 ± 3.5	23.6 ± 3.3	22.3 ± 3.5	< 0.0001
Radiographic HV in at least one foot	766 (39.0%)	149 (22.8%)	617 (46.0%)	< 0.0001
Radiographic HV in both feet	423 (21.2%)	67 (10.2%)	356 (26.5%)	< 0.0001

*Abbreviations*: *HV* hallux valgus

^a^Significantly higher proportion on comparison between men and women, as detected on the chi-square test and subsequent adjusted residual analysis

The radiographic parameters of the 3992 ft and their sex-based differences were summarized in Table 2. The radiographic HV was found in 29.8% (1189 out of 3992 ft), with a significant difference between men and women (16.5% vs. 36.3%, *p* <  0.0001). The mean HVA was 16.8 ± 7.6°, with a significant difference between men and women (14.4 ± 6.2° vs. 18.0 ± 7.9°, *p* <  0.0001). The significantly higher value of HVA in women compared to men was consistent even when separated by age brackets. The mean IPA was 15.4 ± 5.6°, with significantly higher values in men than in women (16.6 ± 4.8° vs. 14.9 ± 5.8°, *p* <  0.0001). The significantly higher value of IPA in men compared to women was limited in those over 50 years of age, when separated by age brackets. A moderate negative correlation between HVA and IPA was found (r = − 0.4308, *p* <  0.0001). The IPA accounted for 48.9 ± 17.6% of TVDH in average, with a significantly higher contribution in men compared to women (54.5 ± 14.6% vs. 46.2 ± 18.3%, *p* <  0.0001). The mean IMA was 8.6 ± 2.6°, with significantly higher values in men than in women (8.0 ± 2.5° vs. 8.9 ± 2.7°, *p* <  0.0001). The significantly higher value of IMA in men compared to women was found in those over 40 years of age except for those aged over 80 years, when separated by age brackets. Moderate correlations between HVA and IMA and between IMA and IPA was found (*r* = 0.5933, *p* <  0.0001; *r* = − 0.4228, *p* <  0.0001; respectively). With respect to HV severity, 1308 ft in men were classified as follows: normal, 1092 ft (83.5%); mild, 188 ft (14.4%); moderate, 22 ft (1.7%); and severe, 6 ft (0.5%). Similarly, 2684 ft in women were classified as follows: normal, 1711 ft (63.7%); mild, 740 ft (27.6%); moderate, 200 ft (7.5%); and severe, 33 ft (1.2%). HV severity had a higher prevalence of normal grade in men, and a higher prevalence of mild, moderate, and severe grades in women.

**Table 2:** Comparisons of radiographic measurements, prevalence of radiographically-assessed hallux valgus and self-recognition about hallux valgus, and grades of hallux valgus severity between feet of men and women

		Total (*n* = 3992)	Men (*n* = 1308)	Women (*n* = 2684)	*P*-value
HVA (degrees)	All	16.8 ± 7.6	14.4 ± 6.2	18.0 ± 7.9	< 0.0001
	Age < 39	15.3 ± 5.0	13.6 ± 4.7	16.7 ± 4.8	0.0004
	Age 40–49	16.4 ± 6.1	14.1 ± 5.3	17.9 ± 6.2	< 0.0001
	Age 50–59	16.5 ± 6.6	13.7 ± 5.0	17.6 ± 6.8	< 0.0001
	Age 60–69	17.0 ± 7.5	14.7 ± 5.9	18.2 ± 7.9	< 0.0001
	Age 70–79	17.1 ± 8.4	14.6 ± 7.0	18.1 ± 8.7	< 0.0001
	Age 80≦	16.6 ± 8.7	14.4 ± 7.2	17.9 ± 9.2	< 0.0001
IPA (degrees)	All	15.4 ± 5.6	16.6 ± 4.8	14.9 ± 5.8	< 0.0001
	Age < 39	17.3 ± 4.2	18.0 ± 3.8	16.7 ± 4.4	0.0501
	Age 40–49	16.6 ± 4.5	16.4 ± 4.6	16.7 ± 4.5	0.6864
	Age 50–59	16.3 ± 4.7	17.1 ± 4.5	16.0 ± 4.8	0.0016
	Age 60–69	15.0 ± 5.5	16.5 ± 4.9	14.3 ± 5.7	< 0.0001
	Age 70–79	14.9 ± 6.1	16.4 ± 4.9	14.2 ± 6.4	< 0.0001
	Age 80≦	15.0 ± 6.2	16.1 ± 5.2	14.3 ± 6.7	0.0007
IMA (degrees)	All	8.6 ± 2.6	8.0 ± 2.5	8.9 ± 2.7	< 0.0001
	Age < 39	7.6 ± 1.7	7.4 ± 1.8	7.8 ± 1.6	0.1787
	Age 40–49	8.2 ± 2.1	7.9 ± 2.0	8.4 ± 2.1	0.0175
	Age 50–59	8.1 ± 2.3	7.6 ± 2.1	8.3 ± 2.3	0.0002
	Age 60–69	8.6 ± 2.6	7.9 ± 2.3	8.9 ± 2.7	< 0.0001
	Age 70–79	9.1 ± 2.9	8.3 ± 2.8	9.5 ± 2.9	< 0.0001
	Age 80≦	9.1 ± 2.9	8.8 ± 2.9	9.2 ± 2.8	0.1432
Contribution of IPA to TVDH (%)		48.9 ± 17.6	54.5 ± 14.6	46.2 ± 18.3	< 0.0001
HV grade (feet [%])	Normal	2803 (70.2%)	1092 (83.5%)^a^	1711 (63.8%)	< 0.0001
	Mild	928 (23.2%)	188 (14.4%)	740 (27.6%)^a^	
	Moderate	222 (5.6%)	22 (1.7%)	200 (7.5%)^a^	
	Severe	39 (1.0%)	6 (0.5%)	33 (1.2%)^a^	
Prevalence of radiographic HV (feet [%])		1189 (29.8%)	216 (16.5%)	973 (36.3%)	< 0.0001
Prevalence of HV self-recognition (feet [%])		657 (16.5%)	71 (5.4%)	586 (21.8%)	< 0.0001

*Abbreviations*: *HVA* hallux valgus angle, *IPA* interphalangeal angle, *IMA* intermetatarsal angle between 1st and 2nd metatarsals, *TVDH* total valgus deformity of the hallux, *HV* hallux valgus

^a^Significantly higher proportion in the comparison between men and women which was detected from the chi-square test and subsequent adjusted residual analysis

**Fig. 2:**
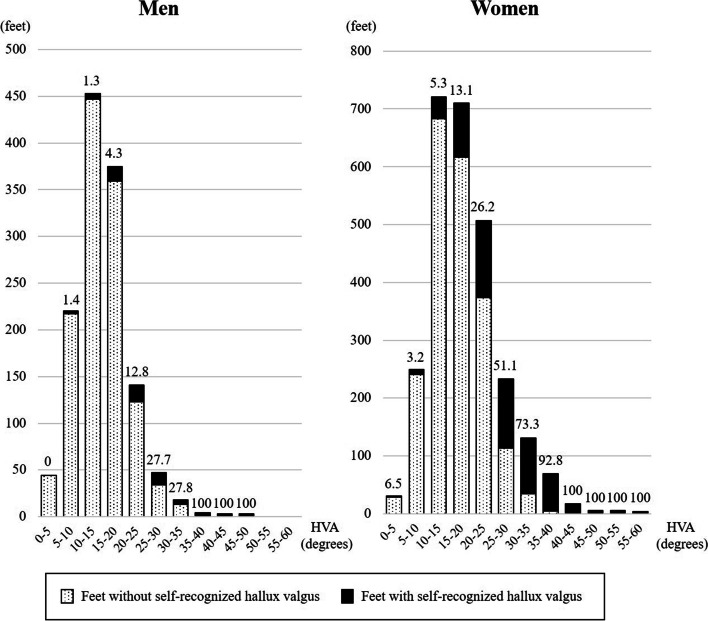
Histograms of the hallux valgus angle in feet with and without self-recognized hallux valgus. The number above each bar indicates the percentage of feet with self-recognized hallux valgus. HVA; hallux valgus angle

The percentage of feet recognized subjectively as HV by participants was 16.5% (657 out of 3992 ft) overall, which was significantly lower than the prevalence of radiographically-assessed HV (*p* <  0.0001). A significant difference in the percentage of HV self-recognition between men and women was observed (5.4% vs. 21.8%, p <  0.0001). The percentage of HV self-recognition increased with an increase in HVA or HV severity (Fig. 2 and Table 3). Among the feet subjectively recognized as HV, 7 out of 71 ft (9.9%) in men and 130 out of 583 ft (22.3%) in women were accompanied by pain (Table 3). The participants reported recognizing HV an average of 17.3 years ago (range, 1–70 years) (Table 3). Among the 409 participants with HV self-recognition, 48 (11.7%) had a history of visiting the hospital for HV, and one (2.4%) had a history of surgery for HV.

**Table 3:** Comparisons of participants’ characteristics, radiographic measurements, and grades of hallux valgus severity between feet with and without self-recognition about hallux valgus

	Men		*P*-value	Women		*P*-value
	Self-recognition			Self-recognition		
	Yes (*n* = 71)	No (*n* = 1237)		Yes (*n* = 586)	No (*n* = 2098)	
Age (years)	67.6 ± 11.2	63.6 ± 13.8	**0.0183**	65.9 ± 10.6	64.0 ± 12.5	**0.0006**
Height (cm)	166.1 ± 7.6	167.2 ± 6.6	0.1824	153.0 ± 6.6	153.7 ± 6.3	**0.0367**
Body weight (kg)	64.6 ± 11.1	66.1 ± 11.1	0.2811	52.6 ± 8.3	52.6 ± 9.2	0.9934
Body mass index (kg/m^2^)	23.4 ± 3.6	23.6 ± 3.3	0.6486	22.5 ± 3.1	22.3 ± 3.6	0.2934
HVA (degrees)	23.8 ± 9.6	13.8 ± 5.4	**< 0.0001**	26.0 ± 8.9	15.7 ± 5.9	**< 0.0001**
IPA (degrees)	12.9 ± 8.7	16.8 ± 4.4	**< 0.0001**	11.0 ± 7.4	16.0 ± 4.8	**< 0.0001**
IMA (degrees)	11.3 ± 4.0	7.9 ± 2.2	**< 0.0001**	11.1 ± 3.3	8.3 ± 2.1	**< 0.0001**
HV grade (feet [%]^a^)						
Normal	25 (2.3%)	1067 (97.7%)^b^	**< 0.0001**	141 (8.2%)	1570 (91.8%)^b^	**< 0.0001**
Mild	31 (16.5%)^b^	157 (83.5%)		252 (34.1%)^b^	488 (65.9%)	
Moderate	9 (41.0%)^b^	13 (59.0%)		160 (80.0%)^b^	40 (20.0%)	
Severe	6 (100%)^b^	0 (0.0%)		33 (100%)^b^	0 (0.0%)	

*HVA* hallux valgus angle, *IPA* interphalangeal angle, *IMA* intermetatarsal angle between 1st and 2nd metatarsals, *HV* hallux valgus

^**a**^ Percentage was calculated as a ratio between those with or without self-recognition in each grade of HV severity

^b^Significantly higher proportion detected from adjusted residual analysis

The comparison of participant characteristics and radiographic measurements between those with HV self-recognition and those without are summarized in Table 3. Those with self-recognition were significantly older, in both sexes (men: 67.6 ± 11.2 years vs. 63.6 ± 13.8 years, *p* = 0.0183; women: 65.9 ± 10.6 years vs. 64.0 ± 12.5 years, *p* = 0.0006), and height was significantly lower only in women with self-recognition (153.0 ± 6.6 cm vs. 153.7 ± 6.3 cm, *p* = 0.0367) (Table 3). Self-recognized feet had significantly higher HVA, lower IPA, and higher IMA than those that were not self-recognized, regardless of sex (HVA: [men] 23.8 ± 9.6° vs. 13.8 ± 5.4°, *p* <  0.0001; [women] 26.0 ± 8.9° vs. 15.7 ± 5.9°, *p* <  0.0001, IPA: [men] 12.9 ± 8.7° vs. 16.8 ± 4.4°, *p* <  0.0001; [women] 11.0 ± 7.4° vs. 16.0 ± 4.8°, *p* <  0.0001, IMA: [men] 11.3 ± 4.0° vs. 7.9 ± 2.2°, *p* <  0.0001; [women] 11.1 ± 3.3° vs. 8.3 ± 2.1°, *p* <  0.0001).

A multivariable analysis revealed that HVA, IMA, and women were independent positive factors of HV self-recognition (HVA [per 1° increase]: OR, 1.18; 95% CI, 1.15–1.20; *p* <  0.0001, IMA [per 1° increase]: OR, 1.15; 95% CI, 1.09–1.20; *p* <  0.0001, women [vs. men]: OR, 3.47; 95% CI, 2.35–5.18; *p* <  0.0001) (Table 4). On the contrary, IPA was detected as an independent negative factor (OR, 0.95; 95% CI, 0.93–0.97; *p* <  0.0001).

**Table 4:** Multivariable logistic regression for geiself-recognition about hallux valgus

	Odds ratio	95% CI	*P*-value
Age (per year)	1.01	1.00–1.02	0.2209
Height (per cm)	1.01	0.99–1.03	0.2296
HVA (per degree)	1.18	1.15–1.20	**< 0.0001**
IPA (per degree)	0.95	0.93–0.97	**< 0.0001**
IMA (per degree)	1.15	1.09–1.20	**< 0.0001**
Gender			
Men	1.00		
Women	3.47	2.35–5.18	**< 0.0001**

*HVA* hallux valgus angle, *IPA* interphalangeal angle, *IMA* intermetatarsal angle between 1st and 2nd metatarsals, *CI* confidence interval

## Discussion

The present population-based cross-sectional study reported the prevalence of radiographically-assessed and self-recognized HV in approximately 2000 participants and the discrepancy between them. The prevalence of HV self-recognition was almost half that of radiographic HV and increased according to HV severity. The present study also demonstrated that valgus position of the proximal phalanx and female sex, but not interphalangeal HV, were independent positive factors for self-recognition of HV.

The present study demonstrated a 29.8% (1189 out of 3992 ft) prevalence of radiographically-assessed HV in two towns, including a mountainous region and a coastal region, which is similar to the investigation by Nishimura et al. reporting a 22.8% (184 out of 806 ft) prevalence in a single mountainous village from a different prefecture in the same country as ours [14]. While the prevalence of HV varies by population group, ethnicity, geographic location, cultural differences in footwear, or socioeconomic status, methodological differences in investigation could also affect the reported value on prevalence. As for the sampling method, studies covering convenience samples such as people visiting foot clinics with foot problems are more likely to report higher prevalence estimates compared to those studies covering people randomly sampled from the general population [4]. One of the strengths of the present study is that we used population-based cohorts, even though our cohort did not include those who could not visit the survey venue or those who did not agree to the survey, and was thus not perfectly free from selection bias. As for the evaluation methods, the studies using clinical examinations showed a decisively higher prevalence than those using self-reporting measures such as interviews or questionnaires [4]. In a meta-analysis of HV prevalence, Nix et al. reported that only 16% of studies in their review used diagnostic criterion for HV based on radiographically- or clinically-measured angle, and that the larger the size of the studied population, the less likely it was to adopt radiographic evaluation for HV and more likely to adopt self-reporting or visual inspection [4]. To our knowledge, there has been no report on HV prevalence using radiographic angular criteria in community-based studies involving over 1000 subjects.

In dealing with underestimation of HV in self-reporting measures, several tools for self-assessment using standardized photographs or line drawings have been developed [8, 9]. For example, the Manchester scale consisting of standardized photographs of feet with four grades of HV has been demonstrated to have excellent re-test and inter-tester reliability in grading HV and also validated based on radiographs [9, 15]. The sensitivity and specificity of self-assessment of HV using the Manchester scale in dichotomous assessment between present or absent of HV has been reported to be 85 and 88% with use of the examiner assessments as the gold standard [9]. A self-assessment tool described by Roddy et al. using five line drawings with sequential increases in HV angle of 15° also has been shown to have excellent re-test reliability but has not yet been validated based on radiographs [15]. The use of these tools had better be considered when surveys about foot disorders will be planned under such circumstances as postal surveys or medical inspections with difficulties in using radiographically- or clinically-measured angle.

Another problem to be faced when interpreting reports about HV prevalence is the use of the term “bunion” in questionnaires or interviews. The term “bunion” means the bursitis located at the medial of first metatarsal head caused by irritation with footwear and is sharply distinguished form the term “hallux valgus” which is a morphological deformation of the hallux [16]. However, these two terms are often used synonymously and cause discrepancies between surveys. For example, this kind of discrepancy is well presented as a large difference in HV prevalence between two surveys by Adams et al. in 1999 and Dunn et al. in 2004, respectively [5, 17]. Adams et al. reported a 0.9% prevalence of bunions among 63,402 persons in the National Health Interview Survey of the USA using questionnaires about whether the questionees had “trouble with bunions” [17]; on the other hand, Dune et al. reported a 37.1% prevalence of bunion on clinical assessment among 784 randomly-sampled community-dwelling adults in the USA, although they seemed to use the term “bunion” to refer to “hallux valgus” [5]. In Japan, a term corresponding to “bunion” does not exist but a term corresponding to “hallux valgus” does. The condition of bunion is generally stated just as “pain or redness caused by HV”; therefore, our question about “hallux valgus” in the present survey can be regarded as asking simply about “deformity” but not about “symptoms”.

The definition of the normal range of HVA also affects HV prevalence. An investigation in Korea by Cho et al. adopted a definition of HV as HVA > 15°, which was broader than our definition of HVA > 20°, and reported a prevalence as high as 64.7% among community-dwelling subjects aged between 40 and 69 years [18]. If we adopted the same definition of HV as Cho et al., HV prevalence in the present study would be 45.2% in men and 62.7%, which is consistent with their study. The “normal range” of anatomical structures is a terminological conception and often arbitrary. It can be based on the distribution of relevant values among asymptomatic healthy subjects or the general population, prognostic prediction, treatment goals, and so on. HV is asymptomatic in a large part of the population; however, the deformity is clinically considered to be progressive, although there has been no consensus about the cut-off value predicting poor prognosis. Many studies using the HV definition of HVA > 15°in radiographic assessments are based on the study by Hardy and Clapham in 1951, reporting a mean 15.7° of HVA among convenient samples, including the staff and students at college [19]; however, it seems too strict to adopt the mean value as a cut-off value between normal and abnormal values. In the present study, there was a great leap in self-recognition between the two groups with HVA of 15–20° and 20–25° (4.3% vs. 12.8% in men, and 13.1% vs. 26.2% in women); therefore, we consider HVA > 20° to be valuable as one of the borders between normal and abnormal in terms of its influence on body image.

The prevalence of symptomatic HV was as low as approximately 10% in the present study, which might still be an overestimation as the presence of pain from HV was asked only to those participants who had self-recognition of HV. This low prevalence of pain would mean that the majority of HV cases are asymptomatic. Many patients do not visit clinics or seek surgical help until symptoms become troublesome years after they recognize the deformity. A survey of patients aged 20–66 years who were waiting for HV operation at a single hospital reported that 46% of the patients had noticed their deformity before they were 20 years old [19]. A cross-sectional investigation in our cohort consisting of community-dwelling adults demonstrated that participants with self-recognition of HV remembered having their deformity for an average of 17 years, but only 11.7% of them had a history of visiting the hospital for it. HV is generally a progressive deformity that can lead to functional disability and an elevated risk of falls in the elderly [2, 20, 21]. Mild deformity can be managed with conservative treatments such as advice on footwear, exercise, and orthosis in order to decrease pain or prevent progressive deformity [22]; however, symptomatic severe deformity regularly requires surgery. The results of the present study demonstrating low self-awareness of HV especially in mild deformity advocate the need to increase the awareness of foot with mild HV deformity in the general population in order to help promote prevention of HV deformity and prevent subsequent burdens from severe HV deformity.

The HV interphalangeus refers to a laterally deviated distal phalanx of the great toe, which is mainly attributed to its anatomical nature. The IPA was reported to be approximately 13° on average, among 346 British feet [23]. The HV interphalangeus could be presumed to contribute substantially to the total valgus deviation of the hallux, considering that normal HVA, a lateral deviation of the proximal phalanx against the 1st metatarsal, is 5° to 15° [24]. However, the effect of the HV interphalangeus on self-recognition of HV has not been elucidated. Some radiographic studies have described an inverse association between HVA and IPA, which was confirmed in the present study [12, 25, 26]. There are two proposed explanations for this inverse relationship [25, 27]. One explanation is based on the findings from a comparative study of radiographic parameters showing larger IPA in feet with hallux rigidus compared to normal feet or feet in hallux rigidus [27]. The increased stability in the horizontal plane at the metatarsophalangeal joint of hallux rigidus feet would let the laterally diverting force from shoe pressure or muscle activity toward the hallux concentrate at the interphalangeal joint; however, in the unstable metatarsophalangeal joint, these forces would likely affect the metatarsophalangeal joint, leading to HV instead of HV interphalangeus [25, 26]. Another explanation is based on findings from some studies demonstrating a postoperative increase in radiographic measurements of the HV interphalangeus following correction of HV, suggesting possible underestimation of HV interphalangeus due to pronation of the hallux in the foot with HV [28, 29]. A previous study by Strydom was the first to propose the concept of TVDH and evaluate the contribution of IPA to TVDH as 37.9%, which was slightly lower than the contribution of 48.9% found in the present study [12]. This lower contribution of IPA in the study by Strydom et al. might be attributed to the biased cohort of their study consisting of patients who visited clinics for foot problems. Their cohort is assumed to have more subjects with HV than the general population. A possible underestimation of the HV interphalangeus due to pronation of the hallux in the foot with HV has been proposed [29]. In the present study, multivariable analysis was conducted to detect the independent influence of HV interphalangeus on the self-recognition of HV, and IPA was detected as a negative factor for self-recognition, but its effect per unit increase was little compared to that of HVA.

Some limitations of our study must be considered in interpreting the results obtained. First, the present study adopted non-weightbearing foot radiographs instead of weightbearing ones. The use of non-weightbearing radiographs might underestimate the hallux deformity [30], and the discrepancy between self-recognition and radiographic diagnosis of HV might be greater than reported in the present study. Second, the present study was performed in limited areas, including two local towns, and might not reflect the situations in other areas. Although little has been clarified about the influencing factors on body image of HV in the general population, some factors besides those analyzed in the present study, such as educational, environmental, socioeconomic, psychological, and ethnocultural factors, might have the potential to affect self-recognition.

In conclusion, the present cross-sectional study is the first to show a discrepancy between self-recognition and radiographic diagnosis of HV in community-dwelling subjects. Valgus deformity at the metatarsophalangeal joint, metatarsus adductus, and female sex were independent positive factors for the self-recognition of HV, but not for HV interphalangeus. Potentially lowered prevalence of HV should be taken into account when interpreting the data of epidemiological studies using self-reporting based on self-recognition. When planning new studies using self-reporting about the prevalence of HV, the use of self-assessment tools such as standardized photographs or line drawings should be considered in order to avoid underestimation of the prevalence due to the discrepancy between actual deformity and self-recognition.

## Data Availability

All data generated or analyzed during this study are available from the corresponding author upon reasonable request.

## Abbreviations

HV: Hallux valgus
ROAD: Research on Osteoarthritis/Osteoporosis Against Disability
OA: Osteoarthritis
HVA: HV angle
IPA: Interphalangeal angle of the hallux
IMA angle: Intermetatarsal angle between 1st and 2nd metatarsals
TVDH: Total valgus deformity of the hallux
